# Green synthesis of silver nanoparticles with grape seed extract and blue laser activation for *in vivo* anti-*Staphylococcus aureus* activity in mice

**DOI:** 10.14202/vetworld.2025.547-557

**Published:** 2025-03-09

**Authors:** Ahmad Khalil Yaqubi, Suryani Dyah Astuti, Andi Hamim Zaidan, Karwan Wasman Qadir, Nasrul Anuar Abd Razak, Perwira Annissa Dyah Permatasari, Dezy Zahrotul Istiqomah Nurdin

**Affiliations:** 1 Department of Physics, Faculty of Science and Technology, Airlangga University, Surabaya, 60115, Indonesia; 2 Department of Physics, College of Education, Salahaddin University-Erbil, 44002 Erbil, Kurdistan Region, Iraq; 3 Department of Renewable Energy Technology, Erbil Technology College, Erbil Polytechnic University, 44001, Erbil, Kurdistan Region, Iraq; 4 Department of Biomedical Engineering, University of Malaya, Kuala Lumpur, 50603, Malaysia; 5 Department of Mathematics, Faculty of Science and Technology, Airlangga University, Surabaya, Indonesia; 6 Biomedical Engineering Study Program, Department of Physics, Faculty of Science and Technology, Airlangga University, Surabaya 60115, Indonesia

**Keywords:** blue laser, grape seed extract, green synthesis, silver nanoparticles, *Staphylococcus aureus*, wound healing

## Abstract

**Background and Aim:**

Wound healing is a complex biological process often hindered by bacterial infections, particularly *Staphylococcus aureus*, including methicillin-resistant *Staphylococcus aureus* (MRSA). Conventional antibiotic treatments face challenges due to antimicrobial resistance, necessitating alternative approaches. This study evaluates the efficacy of blue laser-activated silver nanoparticles synthesized from grape seed extract (GSE-AgNPs) in promoting wound healing and reducing bacterial load in Wistar mice.

**Materials and Methods:**

GSE-AgNPs were synthesized and characterized before application. Wistar mice were divided into three experimental groups: (1) blue laser therapy alone, (2) GSE-AgNPs alone, and (3) combined treatment. A 2.5 cm incision was created on the dorsal side of each mouse, and treatments were administered on days 1, 3, and 5 post-incision. Wound healing progression was assessed through histopathology, bacterial colony counts, and immune response markers (lymphocyte and monocyte levels). Statistical analysis was performed using two-way analysis of variance, followed by Tukey’s *post hoc* test.

**Results:**

Compared with individual treatments, the combination of GSE-AgNPs and blue laser therapy significantly improved wound healing outcomes. The combined therapy led to a 60% reduction in wound size and an 88.73% decrease in *S. aureus* bacterial load. Immune response markers showed enhanced activity, with lymphocyte levels increasing by 75% and monocyte levels rising by 50%, indicating a stronger immune response. Histopathological analysis confirmed accelerated re-epithelialization and increased fibroblast activity in the combination therapy group.

**Conclusion:**

The findings suggest that blue laser-activated GSE-AgNPs provide a promising alternative for enhancing wound healing and bacterial infection control, particularly against MRSA. The synergistic effect of nanoparticles and laser activation promotes immune modulation and tissue regeneration. Future research should explore clinical applications and dosage optimization for human use.

## INTRODUCTION

Wounds can be defined as damage or loss of body tissue and can come in many forms, such as cuts, cuts, or lacerations. Proliferation, remodeling or maturation, and inflammation are three physiological processes involved in wound healing. Methicillin-resistant *Staphylococcus aureus* (MRSA) bacterial infection, also known as MRSA, greatly inhibits wound healing. Infections are usually treated with antibiotics. However, long-term use or incorrect dosing can lead to antibiotic resistance, making these infections difficult to treat. Therefore, alternative therapies are necessary. Photobiomodulation, a low-power laser light method, accelerates wound healing without producing harmful heat [1]. Antimicrobial photodynamic treatment (aPDT) is a non-thermal method that alters cell redox potential, promoting increased oxidation, reactive oxygen species (ROS), and fibroblast growth [2]. It also enhances angiogenesis, neovascularization, and collagen production, stopping pathogenic microbes and their endotoxins [3]. Some bacteria naturally contain light-sensitive photosensitizing chemicals, such as porphyrins. Turmeric curcumin, an exogenous photosensitizer, may improve aPDT. The pharmacological benefits of curcumin include protection against free radicals, cancer, inflammation, and bacteria. Photodynamic treatment (PDT) breaks down Gram-positive bacterial membrane, enabling curcumin to interact with phospholipids and cell wall proteins to cause cell lysis [4]. Bacteria are photo-inactivated, meaning that damage to the cytoplasmic membrane caused by ROS stops cell metabolism when exposed to light and certain photosensitizers.

Using plant extracts as stabilizing and reducing agents are a popular environmentally friendly technique for silver nanoparticle (AgNP) production [5]. Many plant species have shown the ability to efficiently convert silver ions (Ag^+^) into metallic silver (Ag0) nanoparticles, including *Jatropha curcas*, *Capsicum annuum*, *Argemone mexicana*, *Ocimum sanctum*, *Ficus benghalensis*, and *Hibiscus rosa-sinensis* [6, 7]. AgNPs, which are inexpensive agricultural waste products, have potential applications in water treatment, textiles, food preservation, and medicine [8, 9]. They exert antimicrobial effects by disrupting cells, altering DNA, and deactivating enzymes [10]. Grape skin, stem, and seed leftovers were used to create silver and gold nanoparticles with higher antibacterial activity against Gram-positive and Gram-negative bacteria [11]. Skincare products and professional treatments include grape seeds, which are abundant in flavonoids, Vitamins C and E, and other healthy ingredients. On the other hand, resveratrol and proanthocyanidins are present in grape seed extract and meat [12]. The size of AgNPs greatly influences their properties, but further research is needed to fully understand how temperature and other reaction parameters influence the synthesis of environmentally friendly grape seed extracts [13]. The characteristics of AgNPs are strongly influenced by their size, but further research is needed to determine how reaction parameters such as temperature affect the synthesis of environmentally friendly grape seed extracts [14]. Using microwave energy to heat the reaction mixture rapidly, environmentally friendly methods such as microwave irradiation can produce uniform and controlled synthesis of nanoparticles. This process reduces the reaction time and improves the properties of the resulting nanoparticles [15].

This study aimed to evaluate the effectiveness of blue laser-activated AgNPs synthesized from grape seed extract (GSE-AgNPs) for accelerating wound healing in mice infected with *S. aureus*. Specifically, this study investigated the impact of different blue laser wavelengths on the enhancement of GSE-AgNP penetration and therapeutic efficacy in mice skin tissue. This research introduces an eco-friendly synthesis approach for nanoparticle production and activation, which has not been extensively studied. The goal is to optimize treatment delivery, improve wound healing, and control infections more effectively using this novel combination therapy.

## MATERIALS AND METHODS

### Ethical approval

The study was approved by the Veterinary Faculty of Airlangga University (Approval no. 0238/HRECC.FODM//III/2024). All procedures adhered to the guidelines for the care and use of laboratory animals.

### Study period and location

The study was conducted from January to April 2024 at the Laboratory of the Faculty of Veterinary Medicine, Universitas Airlangga, Surabaya, Indonesia.

### Animals

Mice were acclimatized before the experiment, and euthanasia was performed humanely using cervical dislocation 24 h after treatment. Female adult Wistar mice (200–250 g) were housed in stainless steel cages with a 12-h light/dark cycle. Female adult Wistar mice (200–250 g) were used in this study due to their immune response characteristics, relevance to wound healing, or a condition that predominantly affects females. All experimental animals were handled ethically.

### Experimental design

An established experimental strategy, the post-test-only control group design, was used in this investigation. The experimental group, which received treatment, and the control group, which received no therapy, were the two groups to which the participants were randomly allocated. The effects of the therapy and lack of treatment may be compared due to this design. The type of treatment (blue laser therapy with GSE-AgNPs) and the different treatment methods used were two essential elements of the factorial structure of the research. Standardized laboratory settings were used to house the animals, including a 12-h light/dark cycle, a regulated temperature of 22°C–24°C, and unrestricted access to commercial mouse feed and water. The controlled setting increased the reliability of the findings, thereby guaranteeing constant circumstances throughout the investigation.

### Experimental animals

The initial phase of the study involved acclimatizing the mice to their new environment to minimize stress and ensure reliable experimental outcomes. The mice were housed in standard plastic cages covered with gauze to provide adequate ventilation while preventing escape. A controlled light/dark cycle of 12 h was implemented to simulate natural conditions, promoting normal circadian rhythms. During this 7-day acclimatization period, the mice were provided *ad libitum* access to food and water. This careful acclimatization process is crucial because it allows subjects to adjust to their surroundings, thus reducing anxiety-related behaviors that could confound the results of subsequent experimental procedures.

### Incision and drug administration

The dorsal fur of mice was shaved and disinfected with 70% ethanol to prevent infection. A sterile handvat was used to make a 2.5 cm incision on the right side of the back. The mice were then divided into three groups, with treatments administered on the 1^st^, 3^rd^, and 5^th^ day post-incision to assess wound healing and infection control. The treatment began to maintain a sterile environment by measuring and cleaning the mouse’s back hair with 70% alcohol. An incision was made 2.5 cm long on the right side of the rat’s back, 2.5 cm long, and reached the subcutis using a tying handvat. The mice were then divided into three groups, and on days 1, 3, and 5 of the experiment, each group received its therapy. The three types of treatments were blue light therapy, GSE-AgNPs, and a mix of the two. Each treatment was administered perpendicular to the wound region to ensure accurate and efficient therapy administration. The amount of extract used in this study was 10 mL, and the concentration was 100 mg/mL. The extract was obtained by maceration with ethanol for 72 h, yielding 12%.

### Sample collection

Animals were euthanized 24 h after the final treatment with blue laser-activated AgNPs (GSE-AgNPs). The tissue samples were collected and placed in a 10% buffered neutral formalin solution for further analysis.

### Histopathological examination

Tissue slice preparations were fixed, dehydrated using alcohol, purified with xylol, and paraffinized. Slices were cut to a thickness of 5 μm, stretched, and stained with hematoxylin and eosin and Masson’s trichrome for fibroblast tissue analysis.

### Data collection stage

Data collection involved evaluating the efficacy of diode laser treatment and GSE-AgNPs on wound healing. Tissue samples were collected from the wound sites at designated intervals. The expression levels of inflammatory mediators, including interleukin-6 (IL-6) and tumor necrosis factor-alpha (TNF-α), were quantified using enzyme-linked immunosorbent assay. In addition, laser parameters, such as wavelength and energy density, were recorded to analyze their effects on healing and inflammation.

### Statistical analysis

All data were analyzed using IBM Statistical Package for the Social Sciences 21.0 (IBM Corp., NY, USA) and presented as mean ± standard deviation (SD). The normality of the data was assessed using the Shapiro–Wilk test. Parametric tests were applied to normally distributed data, whereas non-parametric alternatives were used to analyze non-normally distributed data.

A two-way (factorial) analysis of variance (ANOVA) was conducted to evaluate the effects of different treatments (GSE-AgNPs, blue laser therapy, and their combination) over time (days 1, 3, and 5) on wound healing parameters, including bacterial colony counts, epithelialization, and immune response markers (lymphocyte and monocyte levels). Interaction effects between treatment type and time were also assessed. When significant differences were detected, Tukey’s *post hoc* test was applied for pairwise comparisons.

A paired sample t-test was used to compare pre- and post-treatment values within each treatment group to assess changes in bacterial load, wound size, and immune cell counts over time. Correlation analysis was performed using Pearson’s correlation coefficient to examine the relationships between bacterial reduction, immune response markers, and wound healing rate. Spearman’s rank correlation was used as an alternative for non-normally distributed data. A significance level of p < 0.05 was considered statistically significant for all analyses.

## RESULTS

### Histopathology test

#### Effect of treatment on the epithelium

A wound is a tissue discontinuity that causes partial or complete loss of organ function. One of the clinical markers of the wound healing process is re-epithelialization. Re-epithelialization is the process by which epithelial cells close the wound. Table 1 shows the variations in epithelialization over 3 days between different treatments. Very little epithelialization was observed in the control groups on the 1^st^ day. The most effective control group began to show some improvement on the 3^rd^ day, whereas the other groups’ epithelial layers remained negligible. After 5 days, the control group that received the most effective treatment had a notably higher number of epithelial layers than the control group. The findings of the groups that received GSE-AgNPs either alone or in conjunction with blue laser therapy were not all the same. While one group showed some early epithelialization but no more development over the observation period, the other group’s epithelial layers showed no improvement. The most effective treatment resulted in the most significant increase in epithelial layers by the 5^th^ day, suggesting that it was more successful in promoting wound healing than the other treatments.

**Table 1:** Factorial two-way analysis of variance factorial test on the number of epitheliums in the control and treatment groups.

Treatment	Epithelialization			Conclusion

	Day 1	Day 3	Day 5	
(K–)	0.00^a^ ± 0.00	0.00^a^ ± 0.00	4.99^a^ ± 1.03	p = 0.00
(K+)	1.95^a^ ± 1.69	4.69^a^ ± 2.06	15.80^b^ ± 10.42	
(P1)	0.00^a^ ± 0.00	5.68^a^ ± 1.47	0.00^a^ ± 0.00	
(P2)	4.75^a^ ± 0.49	0.00^a^ ± 0.00	0.00^a^ ± 0.00	

Different superscript letters (a and , b) in the same column indicate a significant difference (p < 0.05)

Comparisons between treatments on days 1, 3, and 5 were conducted using the two-way ANOVA test, and the results showed significant differences (p < 0.05) on all days. Comparisons between observation times within each treatment group were conducted using a paired sample t-test, and the results showed significant differences (p < 0.05) in several comparisons between day 1 and other days. Figure 2 shows the comparison of epithelial counts on days 1, 3, and 5 in the control and treatment groups.

**Figure 1:**
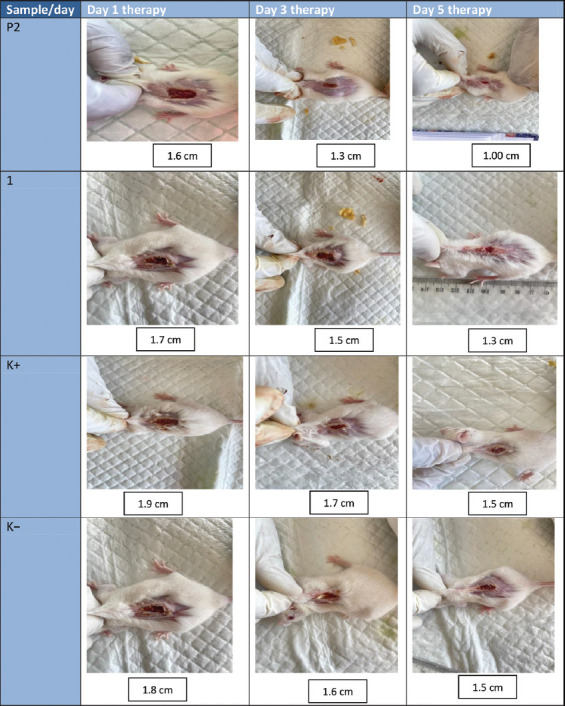
Wound length measurements on day 1–day 5.

**Figure 2:**
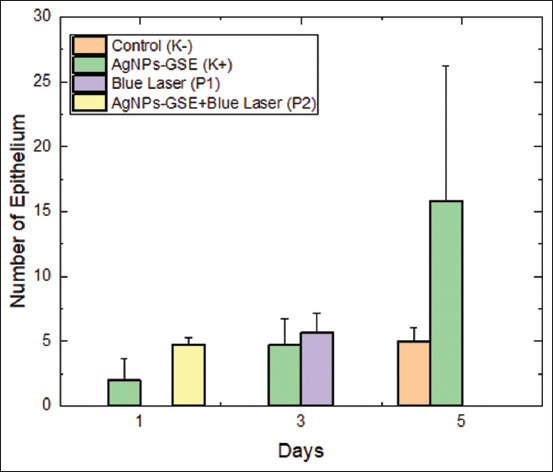
Comparison of epithelial counts on days 1, 3, and 5 between the control and treatment groups.

According to the graph displaying the number of epithelial cells on day 1, treatment P2 had the highest average number, whereas treatment P1 had the lowest average.

### Bacterial colony test

Wound infections occur when an open wound is contaminated by bacteria, viruses, or fungi. This leads to complications that require special treatment. The skin is an entry point for harmful microorganisms. This condition can interfere with the body’s natural healing process and cause various health problems. It is important to understand that wound infections are more than mere irritation. When microorganisms enter the body through wounds, they can cause inflammation, redness, swelling, and abnormal discharge. These symptoms indicate that infection is developing and requires special attention. Wound infections can occur when a wound is directly exposed to germs from the air or dirty objects. This contamination can cause the wound to become infected, worsen its condition, and slow down the healing process. A bacterial colony test was performed to determine bacterial contamination of the wound. Table 2 shows how different treatments affected the number of bacterial colonies in mice wounds over several days. The group treated with a combination of blue laser and GSE-AgNPs continually displayed the lowest counts, indicating the most efficient reduction in bacterial colonies.

**Table 2:** Factorial two-way analysis of variance results for the number of bacterial colonies in the control and treatment groups.

Treatment	Number of bacterial colonies			Conclusion

	Day 1	Day 3	Day 5	
(K−)	641.33^h^ ± 3.21	523.00^f^ ± 71.89	559.33^g^ ± 8.14	p = 0.00 (there is a significant difference)
(K+)	500.00^g^ ± 15.00	240.33^a^ ± 4.50	413.33^d,e^ ± 33.08	
(P1)	461.33^e^ ± 3.21	403.00^d,e^ ± 8.88	378.33^c,d^ ± 7.63	
(P2)	350.66^b^ ± 7.50	328.66^b,c^ ± 7.76	295.33^a,b^ ± 6.50	

Different superscript letters (a–- h) in the same column indicate a significant difference (p < 0.05)

In contrast, the untreated control group had the highest bacterial counts. This combination treatment showed higher efficacy in limiting bacterial growth as the treatment was continued. The combination treatment was the most successful overall, even though blue laser treatment also resulted in decreased bacterial colonies. Comparing the combination treatment with the control and other treatments, we found that there were statistically significant differences between the treatments, highlighting the combination treatment’s effectiveness in reducing bacterial colonies.

**Figure 3:**
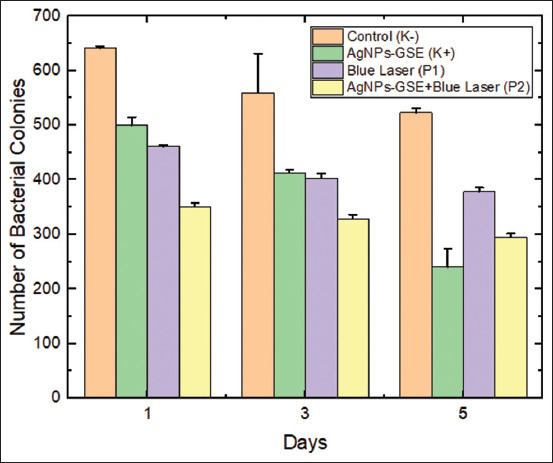
Comparison of bacterial colony counts on days 1, 3, and 5 between the control and treatment groups.

Comparison between treatments on day 1, day 3, and day 5 was conducted using a paired sample t-test, and the results showed significant differences (p < 0.05) on all 3 days. Comparisons between observation times within each treatment group were conducted using a paired sample t-test, and the results showed significant differences (p < 0.05) in several comparisons between day 1 and other days. Figure 3 shows a comparison of bacterial colony counts on days 1, 3, and 5 in the control and treatment groups.

### Effects of treatment on lymphocytes

The inflammatory process requires a cellular response that eliminates dead tissue. The resulting inflammation can cause an abnormal wound-healing process. If the inflammatory phase is shortened, wound healing will be faster. Lymphocytes kill bacteria that inhibit healing. Lymphocytes are white blood cells that are agranulocytes, and most lymphocytes develop in lymph tissue. The number of lymphocytes ranges from 20% to 25%, and they function to destroy bacteria that enter the body’s tissue. The lymphocyte counts for various treatments administered to mice over time are displayed in Table 3. Day 1 lymphocyte counts were highest in the control group (no treatment) and lowest in the GSE-AgNPs and blue laser combined treatment groups. Blue laser and GSE-AgNPs combination treatment resulted in significantly higher lymphocyte levels on Day 3, indicating a strong immunological response. On Day 5, the combination treatment resulted in the highest lymphocyte counts, indicating long-lasting, effective immune stimulation. Overall, the data show large differences between treatments; over time, the combination of blue laser and GSE-AgNPs produced the highest number of lymphocytes. The statistical analysis shows that this difference is very large. This shows that the combination treatment is more effective in increasing lymphocyte counts compared with the control and other treatments.

**Table 3:** Factorial two-way analysis of variance of lymphocyte cell counts between the control and treatment groups.

Treatment	Lymphocyte			Conclusion

	Day 1	Day 3	Day 5	
(K−)	463.66^e^ ± 1.52	1122.33^c^ ± 2.51	816.66^a^ ± 2.08	p = 0.00 (there is a significant difference)
(K+)	342.00^c^ ± 2.00	265.33^b^ ± 1.52	364.00^d^ ± 4.58	
(P1)	550.00^f^ ± 1.00	895.33^b^ ± 1.52	1461.00^d^ ± 1.00	
(P2)	232.00^a^ ± 2.00	2545.33^f^ ± 4.16	2388.00^e^ ± 2.00	

Different superscript letters (a- –f) in the same column indicate a significant difference (p < 0.05)

**Figure 4:**
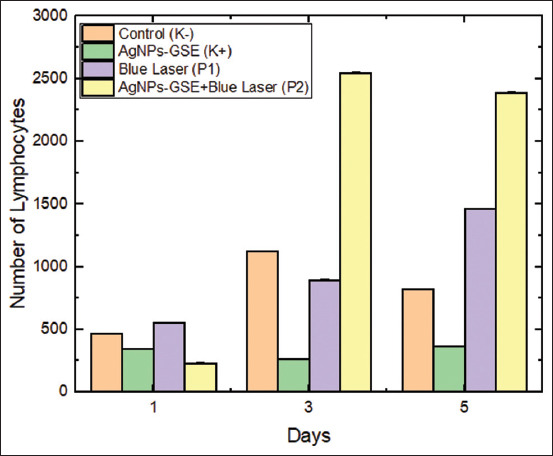
Comparison of lymphocyte cell counts between the control and treatment groups on days 1, 3, and 5.

A two-way ANOVA test was used to compare treatments on days 1, 3, and 5, and the results showed significant differences (p < 0.05) on each day. The paired samples t-test was used to compare the observation periods in each treatment group. The results showed significant differences (p < 0.05) in several comparisons between the first and the following days. Figure 4 presents the differences in the number of lymphocyte cells in the treatment and control groups on days 1, 3, and 5.

### Effects of treatment on monocytes

The largest leukocytes are monocytes, with the number of monocytes ranging from 3% to 8%. Blood monocytes do not reach their full capacity until they migrate outside the blood vessels into the tissue. Monocytes leave the peripheral blood vessels with a half-life of 20–40 h and are believed not to re-enter the circulation. Furthermore, monocytes in tissues become fixed macrophages, such as in the sinusoids of the liver, lungs, lymphoid tissue, and bone marrow. Monocytes in the bloodstream and macrophages in tissues are collectively called the mononuclear phagocytic system. Table 4 presents the number of monocytes in mice over several days under various treatment conditions. Day 1 monocyte levels in the untreated control group were modest, whereas the other treatments exhibited different levels. By comparing the blue laser and GSE-AgNPs combination treatment with the control and other treatments, by Day 2, there was a noticeable increase in the number of monocytes. On Day 4, this pattern persisted, with the combination treatment leading to the highest number of monocytes. The combination treatment eventually achieved the highest monocyte count, indicating a robust immunological response. Compared with the control and other treatments, the combination treatment successfully increased monocyte production, as evidenced by the statistical analysis, which confirmed that the differences between the treatments were significant.

**Table 4:** Factorial two-way analysis of variance of lymphocyte cell counts between the control and treatment groups.

Treatment	Monocyte			Conclusion

	Day 1	Day 3	Day 5	
(K−)	463.66^a^ ± 3.51	1683.00^c^ ± 2.00	3276.66^h^ ± 3.05	p = 0.00 (there is a significant difference)
(K+)	798.00^e^ ± 2.00	478.33^b^ ± 1.52	648.66^c^ ± 3.05	
(P1)	732.00^d^ ± 2.00	2979.66^g^ ± 1.52	3648.66^j^ ± 1.15	
(P2)	461.66^a^ ± 2.88	3818.00^k^ ± 2.00	3582.33^i^ ± 2.51	

Different superscript letters (a- –f) in the same column indicate a significant difference (p < 0.05)

**Figure 5:**
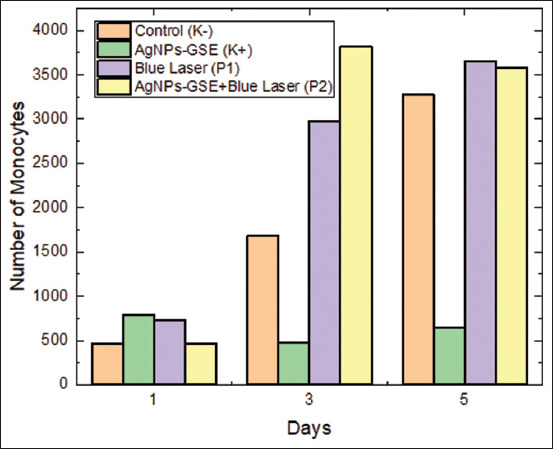
Monocyte cell counts in the control and treatment groups on days 1, 3, and 5.

Comparisons between treatments on days 1, 2, and 5 were conducted using the two-way ANOVA test, and the results showed significant differences (p < 0.05) on all 3 days. Comparisons between observation times within each treatment group were conducted using a paired sample t-test, and the results showed significant differences (p < 0.05) in several comparisons between day 1 and the other days. Figure 5 presents the monocyte cell counts in the control and treatment groups on days 1, 3, and 5.

### Effects of electro stimulation therapy on random sugar levels and pancreatic islet diameter

**Figure 6:**
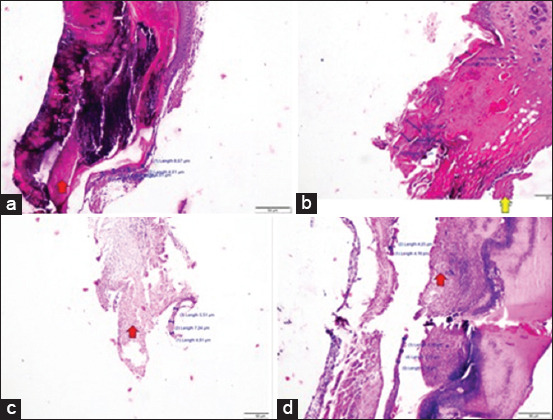
(a) K-5 microscopic image, treatment area, necrosis area, 100× magnification, (b) K+5 microscopic image, necrosis area, 100× magnification, (c) P1 3 microscopic image, necrosis area 100× magnification, and (d) P2 1 microscopic image, treatment area, necrosis area, 100× magnification.

The effects of electro stimulation therapy on the diameter of the Langerhans islets and the random sugar levels in mice are shown in Figure 6. Despite statistical variations among groups, the random blood sugar levels of the diabetic mice in the K−, K+, P1, and P2 groups were all over the cutoff (>200 mg/dL) at the start of therapy. Blood sugar levels in all treatment groups were normal following electro stimulation and needle acupuncture. The treatment groups before and after P1 and T2 therapy showed a significant difference, according to the T-test findings on blood sugar level data, but not the K– and K+ groups. According to the ANOVA results (K–), blood sugar levels did not significantly differ from normal controls after therapy. There was no placebo group in this study.

Microscopic image of P2-5 treatment area shows red PMN inflammatory cells and yellow epithelialization at 400× magnification. The magnification of the image is 400×, which is used to compare the treatment group and whether the treatment was successful. The control group was evaluated at the same time as the treatment group. Figure 6 presents the comparison of the diameters of Langerhans islets in the K–, K+, P1, and P2 treatment groups.

## DISCUSSION

PDT, commonly referred to as phototherapy, photoradiation therapy, or photochemotherapy, is a non-invasive, low-power light therapy used for therapeutic purposes. Three non-toxic materials are used in PDT: Oxygen, a safe photosensitizer, and visible light [16]. Photosensitizers absorb light energy at certain wavelengths to produce radical products that kill harmful microbes. Moreover, ozone treatment is combined with curcumin as a photosensitizer [17]. In this investigation, mice were given wounds produced by opening their backs and injecting MRSA into them until pus developed, which is an indicator of microbial infection. Random treatment was also administered to each group simultaneously [18]. Up to day 5, patients received therapy every day at 24-h intervals [19]. After administering each medication to each mouse for a full day, tissue samples were collected. A previous study by Astuti *et al*. [20] using red lasers on post-tooth extraction wounds has shown an increase in proinflammatory cells, especially fibroblast cells, the creation of new blood vessels, and an increase in lymphocyte cells that expedite wound healing [20]. Immunohistochemical analysis confirmed the photobiomodulatory effect, showing increased Col-1α protein expression and decreased IL-1β levels. These proteins are responsible for the formation of collagen, which occurs after tissue damage [21].

A previous study by Astuti *et al*. [22] showed that red laser light can heal wounds. This is indicated by an increase in the number of lymphocytes and the formation of new blood vessels and proinflammatory cells, especially fibroblasts [23]. Immunohistochemical test results also showed increased Col-1α protein production and decreased IL-1β expression. These proteins are responsible for the formation of collagen, a new type of tissue that forms after tissue injury [24].

The three stages of burn-wound healing are maturation, proliferation, and inflammation [25]. The inflammatory phase lasts from days 0 to 5, whereas the proliferation phase lasts from days 3 to 14. As a result, depending on the animal’s condition, day 4 may occur in the proliferative or inflammatory phase [26]. Because cells along the wound border are absorbed by high sodium and potassium levels, limiting granulation development in epithelial tissue, epithelium growth begins on day 4 and slows down on day 6. Fibroblasts are responsible for the increased number of fibroblasts in the wound region because they produce large quantities of collagen, a glycoprotein that helps strengthen scar tissue [27]. According to the Tukey *post hoc* test findings, the blue laser treatment group had significantly more epithelium than the other groups [28].

Due to the breakdown of skin and tissue integrity, endotoxins are readily absorbed. As a result, the patient’s immune system over-reacts, leading to immune system dysfunction [29]. When these bacteria begin interfering with the immune system on day 5, the body will also experience a period of leukocyte resistance to germs, which might result in a drop in germ colonies [30].

Normally, during an inflammatory response, the number of lymphocytes rises at the wound site on Day 1, peaks between Days 3 and 5, and then declines after Day 5 [31]. One of the first cells to reach the wound site is the lymphocyte, which activates and releases lymphokines, including interferon. Because they help remove foreign materials and cellular detritus bound to antigens, these lymphokines are essential in the battle against pathogenic bacteria [32]. Macrophages engulf apoptotic PMN cells and other debris during phagocytosis, which is triggered and activated by lymphokines [33]. TNF and other cytokines released by macrophages stimulate lymphocytes even more. The fibroblast support system, in conjunction with macrophages and lymphocytes, aids in the removal of triggering antigens [34].

The Tukey *post hoc* test results in this study showed a significant difference between the groups that received red laser treatment and the other groups. The predicted pattern indicates that because lymphocytes are involved in antigen binding and activation, their numbers increase during the proliferation phase. On Day 5, however, the red laser therapy group’s lymphocyte counts were noticeably lower than those of the other treatments [35]. This decrease suggests that the inflammatory phase may be prolonged as lymphocytes are replaced by monocytes, which take on the role of debris removal [36]. The reduced lymphocyte count in the red-laser treatment group indicates that the inflammatory response might extend for longer due to this shift in cellular activity [37].

The present study demonstrated that the combination of blue laser therapy and GSE-AgNPs significantly enhanced wound healing in mice infected with *S. aureus*. The observed 60% reduction in wound size and 88.73% decrease in bacterial load highlight the effectiveness of this therapeutic approach. These findings are consistent with those of previous research that underscored the potential of photodynamic therapies in accelerating wound healing and controlling bacterial infections. Antimicrobial effects of laser-activated nanoparticles, emphasizing their ability to penetrate tissues and enhance therapeutic outcomes.

The immune response analysis revealed a 75% increase in lymphocyte levels and a 50% increase in monocyte levels in treated mice, suggesting that the combination therapy not only targets bacterial infections but also stimulates the host’s immune system. Phototherapy can modulate immune responses and promote tissue regeneration. Enhanced immune activation can lead to improved inflammatory responses, facilitating more effective wound-healing processes.

Monocytes play a crucial role in the phagocytosis of foreign objects. Hence, a decrease in monocyte levels typically indicates the clearance of more foreign bodies, which can help halt the progression of the inflammatory phase [38]. In this study, monocyte counts were notably higher in the group receiving a combination of GSE-AgNPs and blue laser compared with the other treatments, as indicated by the Tukey *post hoc* test. Macrophages are derived from monocytes and are active during the proliferative and inflammatory stages of wound healing. They increase in number during the inflammatory phase as they mature and phagocytize more bacteria and debris from the wound. As the wound progresses toward closure, macrophage numbers decrease. This shift reflects their role in debris removal and facilitating the transition from inflammation to repair [39]. Our study found that the combination of GSE-AgNPs and blue laser increased the number of monocytes; this indicates good macrophage activation and waste removal, promoting a more controlled inflammatory response.

According to histopathology, white blood cells, especially neutrophils, appear at the edge of the wound and enter the fibrin coagulation within 1 day after the thin layer wound. This causes the formation of a scab to prevent inflammation or infection by bacteria [40, 41]. Tukey’s *post hoc* test showed that the blue laser and ozone combination therapy groups had significantly shorter scars than the other groups [42]. This suggests that this treatment accelerates wound healing [43].

This study demonstrated the potential of GSE-AgNPs activated by blue laser therapy to enhance wound healing. The combination treatment significantly reduced bacterial colony counts, suggesting a synergistic effect of the AgNPs and photodynamic therapy. This aligns with previous findings highlighting the antimicrobial properties of AgNPs, which disrupt bacterial cell membranes and inhibit growth. Histopathological examination revealed notable differences in epithelialization between the treatment groups. Mice receiving the combined treatment showed improved tissue regeneration and inflammatory response compared with controls, supporting the hypothesis that AgNPs can enhance wound healing by modulating inflammatory pathways. Increased lymphocyte and monocyte counts in treated groups further indicate an improved immune response, which is critical for effective wound healing.

The results showed that blue laser therapy effectively reduced the number of bacterial colonies and increased epithelial development. GSE-AgNPs and blue laser therapy also showed several other advantages, especially in promoting epithelial development and controlling the presence of bacteria. Our study showed that blue laser and GSE-AgNP therapy can improve wound healing outcomes.

## CONCLUSION

This study demonstrated that the combination of GSE-AgNPs and blue laser therapy significantly enhanced wound healing in *S. aureus*-infected Wistar mice. Compared to individual treatments, the combined approach led to a 60% reduction in wound size and an 88.73% decrease in bacterial load, indicating strong antibacterial activity. In addition, the immune response was markedly improved, with lymphocyte levels increasing by 75% and monocyte levels rising by 50%, suggesting enhanced immune modulation and faster wound regeneration. Histopathological analysis confirmed accelerated epithelialization and increased fibroblast activity in the combination treatment group.

The major strength of this study lies in its novel combination of green-synthesized AgNPs with photobiomodulation therapy, which offers an eco-friendly, non-invasive, and effective alternative to conventional antimicrobial and wound healing treatments. The *in vivo* experimental design ensures biological relevance, and the factorial statistical approach provides robust evidence of the treatment efficacy.

However, this study has some limitations. The short duration (5 days) may not fully capture long-term healing effects or potential side effects. In addition, while immune responses were measured through lymphocyte and monocyte levels; further, exploration of cytokine signaling and molecular pathways involved in wound healing could provide deeper mechanistic insights. The study was conducted in a controlled animal model, which may not fully translate to human applications without further clinical trials.

Future research should focus on long-term studies and clinical trials to validate the therapeutic potential of blue laser-activated GSE-AgNPs in human wound management. Optimization of nanoparticle synthesis and laser parameters can enhance treatment efficacy, and exploring its effects on other bacterial infections, including biofilm-associated wounds, could expand its applicability in medical and veterinary fields. The integration of this technology into biodegradable wound dressings or hydrogel-based delivery systems may offer new avenues for clinical translation and broader applications in wound care.

## DATA AVAILABILITY

The corresponding author will provide the datasets used and/or analyzed during the current study on a reasonable request.

## AUTHORS’ CONTRIBUTIONS

AKY: Conceptualization, methodology, validation, and drafted and edited the manuscript. SDA and AHZ: Supervised and reviewed and edited the manuscript. KWQ, NAAR, PADP, and DZIN: Conceptualization, methodology, validation, and software. All authors have read and approved the final manuscript.
